# The cytotoxic effect of α-tomatine in MCF-7 human adenocarcinoma breast cancer cells depends on its interaction with cholesterol in incubation media and does not involve apoptosis induction

**DOI:** 10.3892/or.2013.2778

**Published:** 2013-10-02

**Authors:** LENKA SUCHA, MILOS HROCH, MARTINA REZACOVA, EMIL RUDOLF, RADIM HAVELEK, LUDEK SISPERA, JANA CMIELOVA, RENATA KOHLEROVA, ALES BEZROUK, PAVEL TOMSIK

**Affiliations:** 1 Department of Medical Biochemistry, Charles University in Prague, Faculty of Medicine in Hradec Kralove, Czech Republic; 2 Department of Medical Biology and Genetics, Charles University in Prague, Faculty of Medicine in Hradec Kralove, Czech Republic; 3 Department of Biological and Biochemical Sciences, University of Pardubice, Faculty of Chemical Technology, Czech Republic; 4 Department of Histology and Embryology, Charles University in Prague, Faculty of Medicine in Hradec Kralove, Czech Republic

**Keywords:** α-tomatine, MCF-7, cell death, cholesterol

## Abstract

In recent years, α-tomatine has been studied for its anticancer activity. In the present study, we focused on the cytotoxic effect of α-tomatine in the MCF-7 human breast adenocarcinoma cell line, its mechanism of action, biotransformation and stability in the culture medium. We observed an inhibition of cell proliferation and viability at concentrations of 6 and 9 μM but then a recovery of cells occurred. The recovery was not caused by the biotransformation of α-tomatine in MCF-7 cells, but by a substantial decrease in the concentration of α-tomatine in the culture medium due to its binding with cholesterol. Regarding the mechanism of action of α-tomatine, we observed no DNA damage, no changes in the levels of the proteins p53 and p21^WAF1/Cip1^, and no apoptosis (neither activated caspase-8 and -9, nor sub-G1 peak, or morphological signs). We found a loss of ATP in α-tomatine-treated cells. These results support the conclusion that α-tomatine does not induce apoptosis in the MCF-7 cell line.

## Introduction

α-Tomatine is a natural steroidal glycoalkaloid, occurring mainly in stems, leaves and roots of tomatoes (*Solanum lycopersicon L*.) and other *Solanum* species. Its content is high in immature green tomatoes and decreases during ripening. α-Tomatine is composed of the 6-ring steroidal aglycone tomatidine by which a tetrasaccharide moiety (containing xylose, galactose and 2 glucose units) is bound to the 3-OH group of the aglycone. Its partial hydrolysis leads to the loss of different sugar parts of α-tomatine and to the formation of β_1_-(β_2_-) tomatine (containing a trisaccharide moiety), γ-tomatine (with a disaccharide) and δ-tomatine (with a monosaccharide) (1,2).

α-Tomatine is a biologically active compound that possesses numerous health-related properties. It exhibits antiviral, antibiotic and anti-inflammatory activity (3–5), stimulates antigen-specific humoral and cellular immune response (6), inhibits acetylcholinesterase activity (7) and has cardiotonic effects (8). Its other effects are connected to its ability to form an insoluble complex with cholesterol in a 1:1 molar ratio (2,9). Since such complex with dietary cholesterol is not able to pass through the intestinal wall, the p.o. administration of α-tomatine decreases the plasma cholesterol level (10). Moreover, α-tomatine disrupts cholesterol containing mammalian biomembranes which could lead to various symptoms of intoxication (gastrointestinal disturbances, haemolysis) (11).

In recent years, the anticancer effect of α-tomatine and its mechanism of action have been studied. In tumor-bearing mice, α-tomatine inhibited tumor growth at doses of approximately 1 mg/kg (12–14). *In vitro*, α-tomatine inhibits the growth of human colon HT29 and liver HepG2 cancer cell lines more than classical anticancer agents (doxorubicin, camptothecin) (15). Furthermore, it is cytotoxic to different human lung cancer cells (A549, NCI-H460) (16,17), human prostatic adenocarcinoma PC3 cells (18), mouse EL4 lymphoma cells (19), several leukemia cell lines (14,20) and breast adenocarcinoma MCF-7 cells (21). Previous studies demonstrated the caspase-independent cell death in α-tomatine-treated human leukemic cell lines (14,20). In the PC-3 cell line, α-tomatine induced apoptosis (18). Furthermore, it inhibited the migration and invasion of cancer cells by inactivating FAK/PI3K/Akt and Erk signaling pathways with a decrease in the binding activity of NF-κB (16,17,21).

In the present study, we examined the dose- and time-dependent inhibition of the growth of the human breast adenocarcinoma MCF-7 cell line after α-tomatine treatment. Due to conflicting reports in the literature regarding the induction of apoptosis by α-tomatine, we also focused on the mechanism of action and DNA damage in MCF-7 cells. The stability of α-tomatine in culture medium and its biotransformation were also studied.

## Materials and methods

### Cell culture and cytostatic treatment

Human breast adenocarcinoma cell line MCF-7 was obtained from the American Type Culture Collection (ATCC; Manassas, VA, USA) and maintained in Dulbecco’s Modified Eagle’s Medium (Sigma-Aldrich, St. Louis, MO, USA) supplemented with a 10% fetal calf serum, L-glutamine, penicillin, streptomycin and non-essential amino acids. Cell cultures were maintained at 37°C in a humidified atmosphere of 5% CO_2_ and the culture medium was renewed every 2–3 days. Cells were detached by incubation with trypsin. Cell lines in a maximal range of up to 20 passages were used for this study. Appropriate amounts of stock solution of α-tomatine (Santa Cruz Biotechnology, Inc., Santa Cruz, CA, USA) dissolved in DMSO were added to the culture medium to achieve the final concentration of 1–9 μM. The control group was treated with DMSO at the concentration of 0.6% in every assay. For irradiation treatment, the cells were irradiated in culture flasks at room temperature using a ^60^Co γ-ray source (Chisotron, Chirana, Ostrava, Czech Republic) at a distance of 1 m from the source, at the photon dose rate of 1 Gy/min.

### WST-1

For assessing the cytotoxic effect of α-tomatine on cell viability, WST-1 (Roche, Basel, Switzerland) reagent was used. MCF-7 cells were seeded in 96-well microtiter plates at a concentration of 5×10^3^ cells/well in 200 μl of culture medium. The cells were allowed to settle overnight at 37°C and in 5% CO_2_. Next, cultures were exposed to different concentrations (1, 3, 6, 9 μM) of α-tomatine for differing time intervals. At the end of each interval, cells were rinsed with PBS and 100 μl of WST-1 was added. Absorbance was measured after 2 h of incubation with WST-1 at 450 nm and a reference wavelength of 650 nm in a Tecan SpectraFluor Plus spectrometer (Tecan Austria GmbH, Grödig, Austria).

### Time lapse videomicroscopy

MCF-7 cells were seeded into plastic tissue-culture dishes with glass bottoms and left for 24 h in an incubator with 5% CO_2_ at 37°C. The next day, the growth medium was replaced with a medium containing different concentrations of α-tomatine (3 and 9 μM). The tissue-culture dishes were transferred into a time-lapse imaging system BioStation IM (Nikon, Prague, Czech Republic) combining an incubator, a motorized microscope and a cooled CCD camera. Recording was carried out in a multipoint and multichannel manner employing various time-lapse modes and upon small as well as high magnifications to allow global as well as detailed view of changes in the behavior of the treated cell populations. Recorded sequences were subsequently semi-automatically analyzed with the software NIS-Elements AR 3.20 (Nikon).

### xCELLigence

Growth of MCF-7 cells was continuously monitored for at least 90 h using the xCELLigence RTCA MP instrument (Roche). The background impedance signal was measured with 50 μl of cell culture medium/well. The final volume in a single well was adjusted to 100 μl of cell culture medium by adding an additional 50 μl of medium containing MCF-7 cells. The impedance was recorded in 15 min intervals. Twenty-four hours after seeding, α-tomatine was added to the culture. All incubations were performed at a volume of 150 μl. α-Tomatine was analyzed at 4 different concentrations (1, 3, 6 and 9 μM), each one with 4 replicates. The impedance signal was analyzed by normalizing data of each single well to the first measurement after starting the treatment: CI_(normalized)_ = CI_time x_/CI_norm time_ (termed here ‘normalized cell index’). This normalized cell index was used for representing the results graphically and exported for further processing using Microsoft Office Excel (Microsoft Inc., Redmond, WA, USA).

### ATP

ATP content in MCF-7 cells exposed to α-tomatine at 4 different concentrations (1, 3, 6 and 9 μM) was measured by an ATP bioluminescent assay kit (Sigma-Aldrich). ATP content was calculated from a standard curve derived from known concentrations of ATP and was expressed as a percentage of the control.

### LDH

LDH activity was determined at individual time points in the medium of α-tomatine exposed MCF-7 cells. An aliquot (0.1 ml) of each sample was added to a cuvette with 0.2 ml of 2.5 mg/ml NADH solution and 0.2 ml of sodium pyruvate solution (1 mg/ml). The total LDH activity was determined after sonication of cells. The enzyme activity was determined spectrophotometrically and the rate of enzyme leakage/min was expressed as percentage of the total LDH activity compared to untreated control cells.

### Analysis of DNA damage (comet assay)

This method is used to assess the single-strand breaks (SSBs) and double-strand breaks (DSBs) present in the DNA. The alkaline and neutral version of the comet assay was used as previously described (22,23). Cells were embedded in 1% agarose (Sigma-Aldrich) on microscope slides and lysed in 10 mM of Tris-buffered 2.5 M NaCl (pH 10.0; Penta, Prague, Czech Republic) with 1% Triton X-100 (Merck KGaA, Darmstadt, Germany), and 100 mM EDTA (Penta) at 4°C for 1 h. The alkaline comet assay (NaOH, EDTA) was carried out at 40 V, 300 mA, for 30 min at 4°C after 40 min of alkaline unwinding. The neutral comet assay (90 mM Tris, 90 mM boric acid, 2 mM EDTA, pH 8.0) was performed at 29 V, 6 mA, for 40 min, at 4°C.

Comets were scored by the Lucia 6.20 image analysis comet module (Laboratory Imaging, Prague, Czech Republic) after staining of cells with ethidium bromide (Sigma-Aldrich). The percentage of DNA in the comet tail was assessed in at least 50 cells/slide and the median DNA damage score for each gel was calculated. Generally, 4–6 gels for each treatment group were scored, and the means ± SD (standard deviation) of medians of these gels are presented.

### Western blot assay

The cells treated with α-tomatine were harvested for the preparation of whole-cell lysates (Cell Lysis Buffer; Cell Signaling Technology, Inc., Boston, MA, USA) and the protein content was quantified using BCA assay (Sigma-Aldrich). Lysates with an equal amount of protein (10 μg) were loaded on 12% SDS-PAGE gels. After electrophoresis, the separated proteins were transferred to a PVDF membrane (Bio-Rad, Hercules, CA, USA). The membranes were blocked in Tris-buffered saline containing 0.05% Tween-20 and 10% non-fat dry milk and incubated with primary antibody (p53; Exbio, Prague, Czech Republic; p53_serine15; Calbiochem-Merck Co., USA; Chk1, Chk1_serine345; Chk2, Chk2_threonine68; Cell Signaling Technology; β-actin, p21^WAF1/Cip1^; Sigma-Aldrich) at 4°C overnight. Then, the membranes were incubated with polyclonal anti-mouse or anti-rabbit secondary antibody (DakoCytomation, Glostrup, Denmark). For band detection, a chemiluminescence detection kit (Roche) was used.

### Caspase activity

In order to detect caspase-8 and -9 activity, Caspase-Glo^®^ Assays (Promega Corporations, Madison, WI, USA) were used according to the manufacturer’s instructions. Briefly, the cells (1×10^4^) treated with the different concentrations of α-tomatine were seeded per well using a 96-well plate format (Sigma-Aldrich) and incubated with 50 μl of Caspase-Glo^®^ Assays reagent for 30 min before the luminescence was measured using a Tecan Infinite M200 spectrometer (Tecan Group, Männedorf, Switzerland).

### Flow cytometry

After 24 and 72 h of incubation with α-tomatine, cells were harvested, washed twice with ice-cold PBS buffer, fixed and permeabilized with 70% ethanol. For detecting low-molecular-weight fragments of DNA, the cells were incubated for 5 min at room temperature in a buffer (192 ml 0.2 M Na_2_HPO_4_ + 8 ml 0.1 M citric acid, pH 7.8) and then stained with propidium iodide in Vindelov’s solution for 60 min at 37°C (all reagents from Sigma-Aldrich). The DNA content was determined by flow cytometer Dako CyAn (Beckman Coulter, Brea, CA, USA) with an excitation wave length of 488 nm; the total emission above 560 nm was recorded. List mode data were analyzed using Multicycle AV software (Phoenix Flow Systems, San Diego, CA, USA) and the percentage of cycling cells was determined.

### Transmission electron microscopy

MCF-7 cells were treated with 6 μM of α-tomatine for 4 h. The cells were fixed in 2% paraformaldehyde and 1% glutaraldehyde in phosphate buffer for 4 h at 4°C. The cells were then post-fixed in 2% OsO_4_ in phosphate buffer for 2 h at room temperature. The dehydration was carried out in graded ethanol and propylene glycol and embedding in Epon-Durcupan (Sigma-Aldrich). Ultra-thin sections (60 nm) were prepared and placed on grids, stained with 2% uranyl acetate solution and 0.2% lead citrate in 0.1 M NaOH, and examined in a Tesla BS-500 transmission electron microscope (Tesla, Brno, Czech Republic). The calibration of magnification was carried out using the calibrator 02902-AB (SPI, West Chester, PA, USA).

### Liquid chromatography, mass spectrometry

#### Analysis of α-tomatine

Separation was performed on a 1200 series liquid chromatography system (Agilent Technologies, Palo Alto, CA, USA) consisting of degasser, quaternary pump, autosampler and thermostated column compartment. The HPLC column used was Accucore C18 (100×2.1 mm, 2.6 μm) (Thermo Scientific, San Jose, CA, USA) held at 40°C. The mobile phase flowing at a rate of 0.6 ml/min consisted of ammonium formate (5 mM, pH=3.8) (solvent A) and acetonitrile (solvent B). The gradient elution program was: from 20 to 27% (v/v) of solvent B in 4.5 min, for 1 min at 50% solvent B and equilibration at 20% (v/v) of solvent B for 2 min.

Column output was coupled with a 3D ion trap LCQ Fleet mass spectrometer with electrospray interface (Thermo Scientific). All mass scans were operated in positive SIM mode at m/z 1033.5–1035.5 for α-tomatine and 883.5–885.5 for solasonine (IS) (Research Plus Inc., Bayonne, NJ, USA) with the following optimized settings for the ion source: spray voltage 4 kV, sheath gas 40 AU, auxiliary gas 10 AU, capillary voltage 20 V, capillary temperature 260°C, tube lens voltage 92 V.

#### Metabolic screening

For metabolite screening, the same chromatographic system and mass spectrometer was used as in the case of α-tomatine analysis. Chromatography was carried out on HPLC column Kinetex C18 (150×3 mm, 2.6 μm) (Phenomenex, Torrance, CA, USA) held at 45°C. The mobile phase flowing at a rate of 0.6 ml/min consisted of ammonium formate (5 mM, pH=3.8) (solvent A) and acetonitrile (solvent B). The gradient elution program was: from 20 to 60% (v/v) of solvent B in 30 min, equilibration at 20% (v/v) of solvent B for 5 min. Mass spectrometric conditions were the same as in the analysis of α-tomatine, data were scanned in full scan mode (150–2,000 m/z).

### Sample preparation for analysis and metabolic screening

#### Acid hydrolysis of α-tomatine

Putative α-tomatine metabolites arising from deglycosidation were prepared by acid hydrolysis of α-tomatine standard. The hydrolysis proceeded in a solution of 0.25 M hydrochloric acid prepared in methanol. α-Tomatine was dissolved at a final concentration of 10 μM in the above-mentioned solution and left 1 h at 60°C. After hydrolysis, 5 μl was injected directly onto the column.

#### Analysis of α-tomatine

Ten microliters of the internal standard (solasonine, 50 μM) and 100 μl of acetonitrile were added to a 100 μl sample aliquot. The sample was vortex mixed, centrifuged (12,700 × g, 10 min, 4°C) and the supernatant transferred to a vial with a glass insert. Five microliters of the sample were injected onto the column.

#### Metabolic screening

Frozen samples (−80°C) of incubation media and MCF-7 cell pellet reconstituted in 100 μl of water were thawed at room temperature. Two hundred microliters of 10% (v/v) formic acid in N,N-dimethylformamide (DMF) were added to a 100 μl sample aliquot. The sample was vortex mixed, centrifuged (12,700 × g, 10 min, 4°C) and transferred to a vial with a glass insert. Five microliters of the sample were injected onto the column. The 10% (v/v) formic acid in DMF was able to solubilize serum proteins, including albumin and to dissociate the cholesterol/α-tomatine complex. In contrast to the precipitation with acetonitrile, the putative metabolites could neither be retained by protein precipitate nor in complexes with cholesterol when 10% (v/v) formic acid in DMF was used for the sample treatment.

#### Statistical analysis

The statistical data for caspase-8 and -9 assays, flow cytometry and comet assay were evaluated with descriptive statistics using Microsoft Office Excel (Microsoft Inc.). Data are expressed as an arithmetic means ± SD of at least 3 independent experiments and statistical analysis was performed using a Student’s t-test. Significant differences were established at p≤0.001.

The statistical data for WST-1, xCELLigence, ATP and LDH assays were evaluated with GraphPad Prism (GraphPad Software version 4.0, Inc., San Diego, CA, USA) software. Data are expressed as an arithmetic means ± SD of at least 3 independent experiments and statistical analysis was performed using one-way ANOVA test and Dunnett’s post-hoc test for multiple comparisons. Significant differences were established at p≤0.05.

## Results

### The cytotoxic effect of α-tomatine in MCF-7 cells

**Figure 1:**
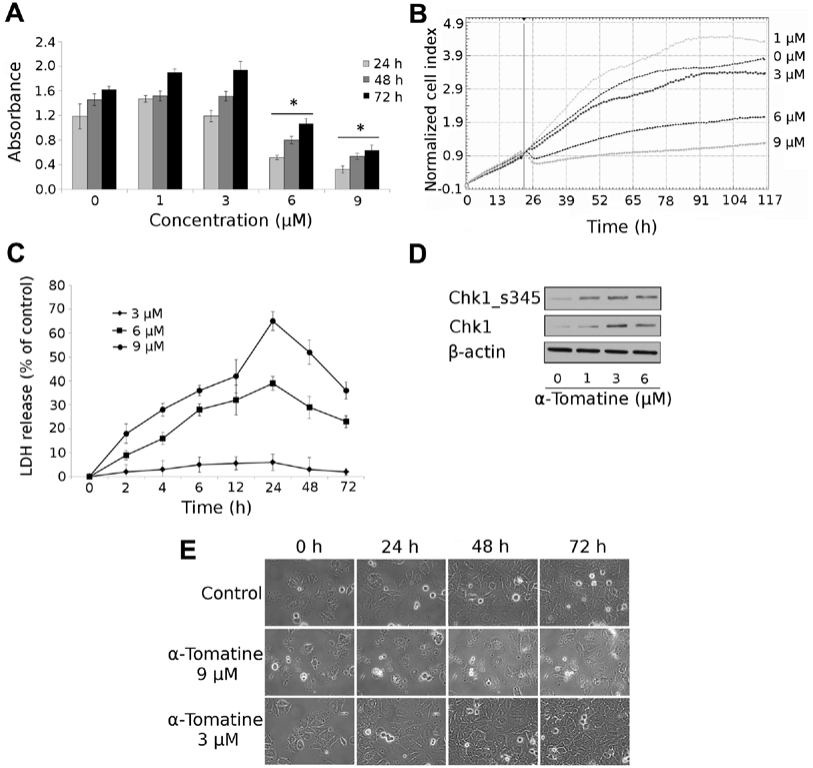
Cytotoxic effect of α-tomatine in human breast adenocarcinoma cell line MCF-7. Cultured cells were treated with α-tomatine at various concentrations for 24, 48 and 72 h, then examined using (A) WST-1 test or (B) xCELLigence assay over 72 h. (C) Cultured cells were treated with α-tomatine (3, 6 and 9 μM) for 2, 4, 6, 12, 24, 48 and 72 h, then LDH release was measured. (D) Induction and activation of Chk1 in MCF-7 cells exposed to 1, 3 and 6 μM α-tomatine 4 h after the application of the drug detected by western blotting. To confirm equal protein loading, membranes were reincubated with β-actin. Chk1_s345 - Chk1 phosphorylated on serine 345. Chk1, check-point kinase 1. (E) Time-lapse videomicroscopy was performed in MCF-7 cells after α-tomatine treatment at various concentrations for 24, 48 and 72 h. Values represent means ± SD of 3 independent experiments (^*^p≤0.05 compared with the untreated control group with one-way ANOVA test and Dunnett’s post-hoc test for multiple comparisons).

We studied the effect of α-tomatine at various concentrations (0, 1, 3, 6 and 9 μM) on cell viability and proliferation of the MCF-7 cell line at different times of exposure (24, 48 and 72 h). A WST-1 assay was used for viability measurement and an xCELLigence system was used to measure proliferation. As shown in Fig. 1A, α-tomatine exerted a dose-dependent inhibitory effect on the viability of MCF-7 cells. Treatment with α-tomatine at concentrations of 1 and 3 μM did not significantly alter the viability of the MCF-7 cell line (compared with the untreated control) after 24, 48 and 72 h. Treatment with 6 and 9 μM caused a significant decrease in the cell viability after 24, 48 and 72 h. Similarly, the proliferation of MCF-7 cells after α-tomatine treatment decreased in a dose-dependent manner and the EC_50_ value after 72 h was 7.17 μM as determined by the xCELLigence system (Fig. 1B). The inhibition of proliferation and viability of MCF-7 cells was the most pronounced after 24 h of α-tomatine action.

Furthermore, we studied the cytotoxic effect on MCF-7 cells using the measurement of LDH release from damaged cells. Again, the concentrations of 6 μM (after 4–72 h) and 9 μM (after 2–72 h) of α-tomatine caused significant damage (Fig. 1C) to treated cells, with the highest effect occurring after 24 h. The release of LDH is a result of membrane destabilization.

The inhibition of proliferation corresponds with the finding of check-point kinase 1 (Chk1) activation. In MCF-7 cells treated with α-tomatine at concentrations of 1, 3 and 6 μM after 4 h, the amount of Chk1 increased. This increase was accompanied by phosphorylation of Chk1 on serine 345 after the same incubation time (Fig. 1D). After 24 h, the changes in Chk1 and its phosphorylated form disappeared (data not shown).

The morphology and behavior of MCF-7 cells after treatment with 3 and 9 μM of α-tomatine were recorded over a 72-h period using time-lapse videomicroscopy. Control cultures showed a time-dependent increase in the number of cells culminating at 72 h, when full confluence was reached. Similar timing and dynamics were also observed upon 3 μM of α-tomatine treatment with no morphological or behavioral alterations. Conversely, 9 μM of α-tomatine significantly reduced proliferation of MCF-7 cells over a 24-h period. Cells did not divide, their membranes demonstrated reduced undulations and many detached from the substratum and rapidly collapsed, releasing their content into the medium. On the other hand, no specific apoptotic morphologies (i.e. blebbing) were observed. In subsequent time intervals (24–72 h of treatment), a number of thus affected cells decreased and the remaining cells started to behave actively including mitoses. At the end of the experiment (72 h), the treated culture clearly recovered and continued in proliferation (Fig. 1E).

These results indicated the significant cytotoxic effects of α-tomatine at concentrations of 6 and 9 μM after 4, 6, 12 and 24 h of treatment. After 48 and 72 h of treatment, the cytotoxic effects persisted, but the cells started to proliferate again and their viability increased.

### Assessment of DNA damage in MCF-7 cells after α-tomatine treatment

**Figure 2:**
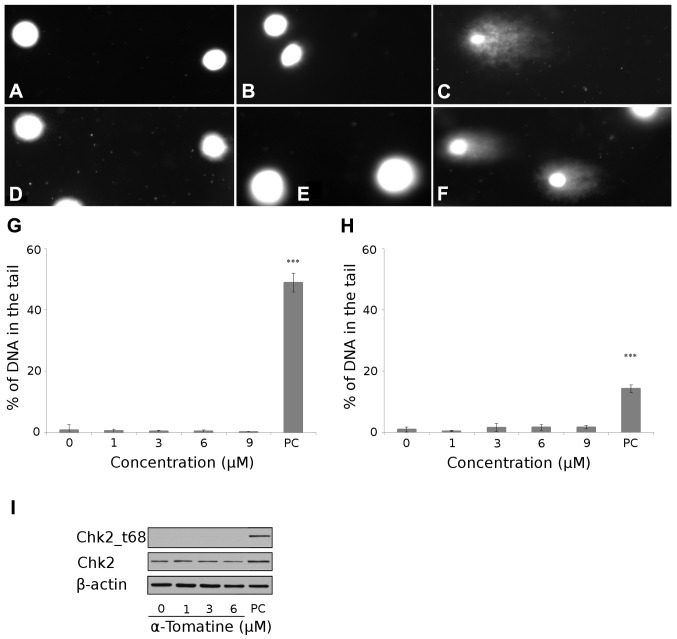
Detection of DNA damage. Digital camera image of the comet assay; the alkaline version (top row), (A) untreated MCF-7 cells, (B) 6 μM of α-tomatine-treated cells and (C) 1.5% H_2_O_2_-treated cells (positive control). Digital camera image of comet assay: the neutral version (bottom row), (D) untreated MCF-7 cells, (E) 6 μM of α-tomatine-treated cells and (F) cells after exposure of 20 Gy of irradiation (positive control). Microscope magnification, ×20. (G) The dependence of DNA single-strand breaks on the concentration of α-tomatine after 4 h of exposure in MCF-7 cells. Asterisks indicate values significantly (p<0.001) different from the control (distinguished using Student’s t-test). PC, positive control (1.5% H_2_O_2_). (H) The dependence of DNA double-strand breaks on the concentration of α-tomatine after 4 h of exposure in MCF-7 cells. Asterisk indicate values significantly (p<0.001) different from the control (Student’s t-test). PC, positive control (20 Gy γ radiation). (I) Changes in Chk2 and Chk2 phosphorylated on threonine 68 (Chk2_t68) detected by western blotting 4 h after exposure to 1, 3 and 6 μM of α-tomatine. To confirm equal protein loading, membranes were reincubated with β-actin. PC, positive control (10 Gy γ radiation after 24 h). Chk2, check-point kinase 2.

Since DNA is the main target of most cytotoxic anticancer drugs, we focused on the induction of DNA damage in MCF-7 cells after α-tomatine treatment. To assess DNA damage in MCF-7 cells, we used the alkaline and neutral version of the comet assay. The alkaline version is employed for the quantification of SSBs of DNA. The concentrations of α-tomatine used (1–9 μM) did not cause any significant SSBs after 4 h compared with H_2_O_2_-treated MCF-7 cells (Fig. 2A–C and G). The neutral version specifically determines DSBs of DNA. The same concentrations of α-tomatine, as they were used in the alkaline version of the comet assay, did not cause any significant DSBs after 4 h of treatment in comparison with irradiated cells (20 Gy after 0.5 h). These results indicated that α-tomatine caused no DNA damage in MCF-7 cells (Fig. 2D–F and H).

In the next step, we confirmed no DSBs of DNA by determining check-point kinase 2 (Chk2) non-activation. After 4 h of α-tomatine treatment (1, 3 and 6 μM), no phosphorylation on threonine 68 of Chk2 occurred in comparison with the positive control group (10 Gy irradiation after 24 h) (Fig. 2I).

### Mechanism of cell death and cell cycle analysis

Although our results indicate no α-tomatine-induced DNA damage in MCF-7 cells, we elucidated whether apoptosis is induced in the cells exposed to α-tomatine by another triggering mechanism.

**Figure 3:**
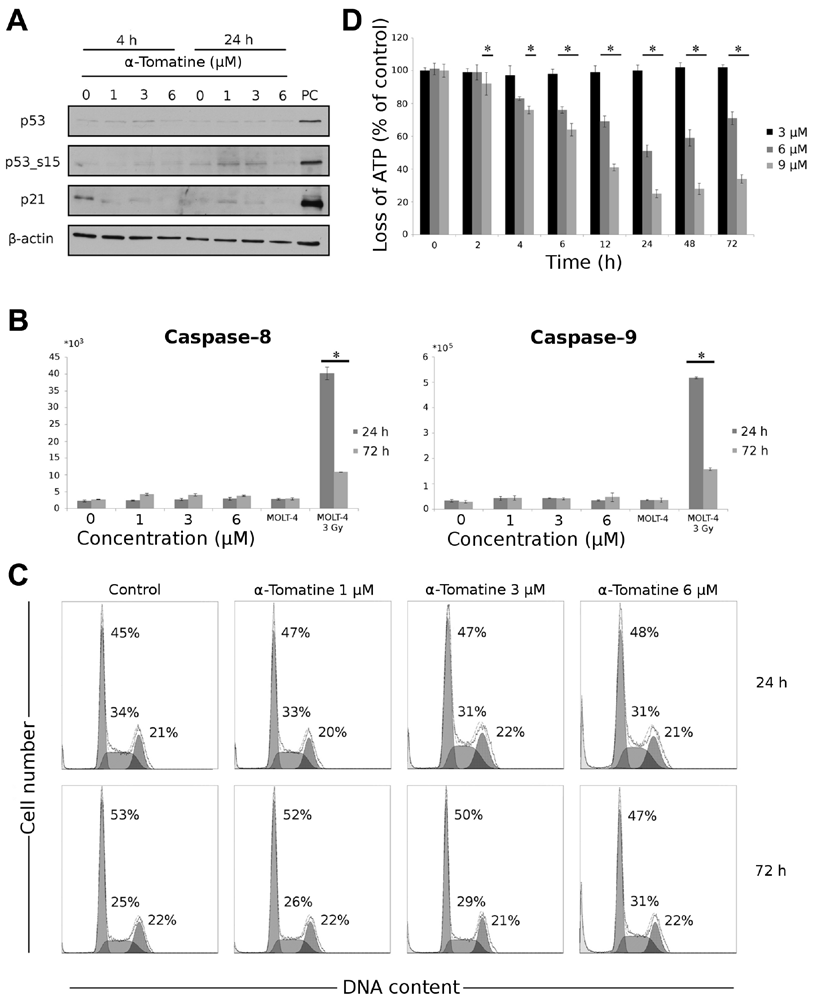
The effect of α-tomatine on the induction of apoptosis in MCF-7 cells. (A) Changes in p53, p53 phosphorylated on serine 15 (p53_s15) and p21^WAF1/Cip1^ detected by western blotting 4 and 24 h after exposure to 1, 3 and 6 μM of α-tomatine. To confirm equal protein loading, membranes were reincubated with β-actin. PC, positive control (10 Gy γ radiation after 24 h). (B) The activity of caspase-8 and -9 was determined 24 and 72 h after exposure to 1, 3 and 6 μM of α-tomatine. γ irradiation (3 Gy) of MOLT-4 cells was used as a positive control. (C) Cell cycle distribution was measured using flow cytometric detection of DNA content in the cells. DNA content was analyzed 24 and 72 h after α-tomatine treatment in concentrations of 1, 3 and 6 μM. (D) The loss of ATP was measured using ATP bioluminescent assay kit at given time intervals after exposure to 3, 6 and 9 μM of α-tomatine. Values represent means ± SD of 3 independent experiments (^*^p≤0.05 compared with the untreated control group with one-way ANOVA test and Dunnett’s post-hoc test for multiple comparisons-loss of ATP and with Student’s t-test-activity of caspases and cell cycle distribution).

Initially, we evaluated changes in the levels of proteins p53, p53 phosphorylated on serine 15 and p21^WAF1/CIP1^ in α-tomatine-treated (1, 3 and 6 μM) MCF-7 cells after 4 and 24 h of incubation. No changes in the levels of these proteins were observed (Fig. 3A).

Then, we measured the caspase-8 and -9 activity after 24 and 72 h of incubation with α-tomatine (1, 3 and 6 μM). No significant increase in the activity of these caspases was observed after α-tomatine treatment (Fig. 3B).

We used cell cycle analysis by flow cytometry to assess possible apoptosis after α-tomatine treatment (1, 3 and 6 μM) for 24 and 72 h. As shown in Fig. 3C, the concentrations of α-tomatine used caused no specific apoptotic sub-G1 peak after 24 and 72 h of treatment.

**Figure 4:**
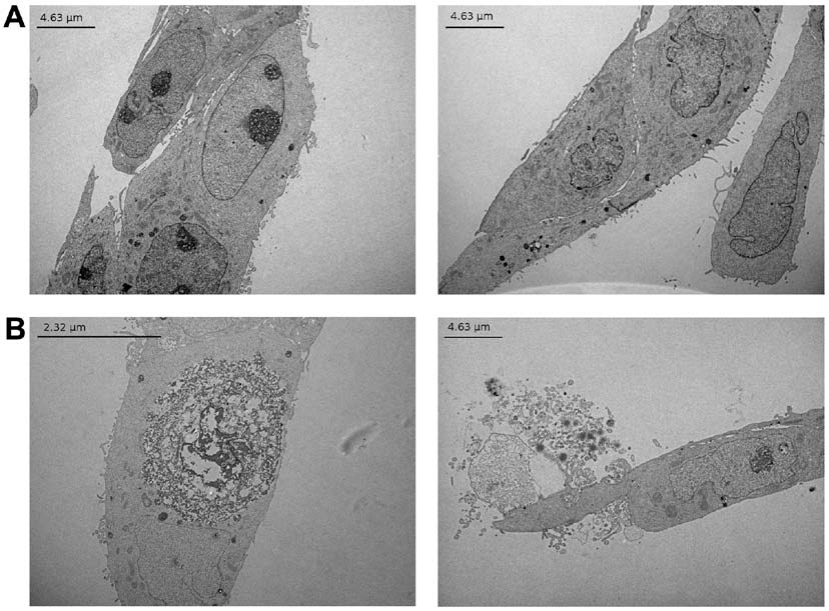
Transmission electron micrographs representative of MCF-7 cells. Confluent cells (60–70%) were treated with 6 μM of α-tomatine for 4 h. Images of transverse sections of the cells are shown. (A) Control cells, (B) α-tomatine-treated cells. The treated cells show disintegration of both the outer and nuclear membranes, but no typical morphologic signs of apoptosis.

To confirm our theory that α-tomatine did not cause apoptosis in MCF-7 cells, we measured the loss of ATP in α-tomatine-treated cells (3, 6 and 9 μM) after 2, 4, 6, 12, 24, 48 and 72 h. Fig. 3D demonstrates a significant decrease, in comparison to untreated cells, in the content of ATP in cells treated with 9 μM of α-tomatine at all treatment intervals between 4 and 72 h and with 6 μM of α-tomatine at all treatment intervals between 6 and 72 h. Moreover, to gain further insight into the mechanism of cell death caused by α-tomatine, transmission electron microscopy of MCF-7 cells treated with 6 μM of α-tomatine was carried out (Fig. 4). The treated cells showed swelling and disintegration of both nuclear and plasma membranes, characteristic of a rapid necrosis, but no typical morphologic signs of apoptosis, such as cell shrinkage, nuclear fragmentation, chromatin condensation, nuclear and cytoplasmic blebbing and the formation of apoptotic bodies.

### Stability of α-tomatine in culture medium and its biotransformation

Previous results indicated that α-tomatine at concentrations of 6 and 9 μM caused a cytotoxic effect in MCF-7 cells during the first 24 h, but after 48 and 72 h of incubation cell proliferation and viability began to increase. Thus, we monitored the concentration of α-tomatine in the medium during the incubation with cells in order to determine whether it changes with time. We detected a decrease in the level of α-tomatine from the initial value of 2.49 to 1.56 μM after 4 h, to 0.90 μM after 24 h, to 0.73 μM after 48 h, and to 0.75 μM after 72 h.

**Figure 5:**
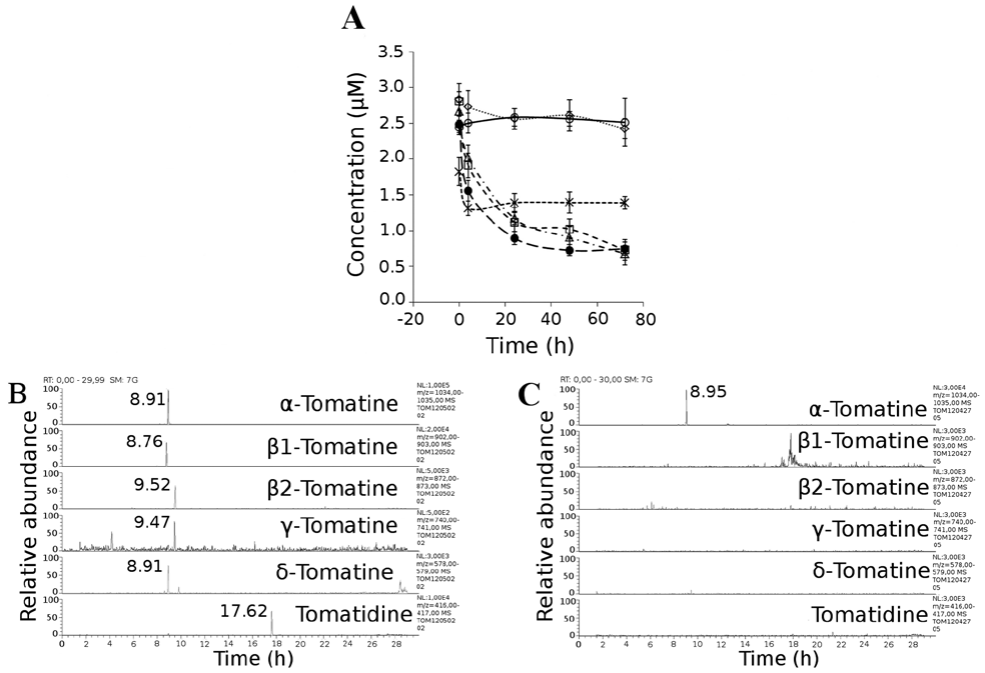
The stability and biotransformation of α-tomatine. (A) 3 μM of α-tomatine was incubated in PBS buffer (**-**⊖**-**), in standard incubation medium with MCF-7 cells (**-**•**-**) and without MCF-7 cells (**-**△**-**), in standard incubation medium, in which 10% fetal calf serum was replaced by 10% inactivated fetal calf serum (55°C, 30 min) (**-**▤**-**), albumin at a concentration of 6 g/l (····⋄····) or albumin at a concentration of 6 g/l and cholesterol at a concentration of 100 μM (--*--). Cells were added only to the standard incubation medium. Samples were obtained at 4, 24, 48 and 72 h of incubation and analyzed by LC-MS in triplicates. The molar ratio cholesterol:α-tomatine was 100:3 in these solutions. (B) Representative LC-MS (ESI+) chromatograms of hydrolysis products of α-tomatine, after acid hydrolysis. (C) Representative LC-MS (ESI+) chromatograms of incubation media after 72 h of incubation of MCF-7 cells with 3 μM of α-tomatine.

As cholesterol is known to form an insoluble precipitate with α-tomatine, its effect in the incubation media was evaluated. Initially, the stability of 3 μM of α-tomatine in PBS buffer (pH 7.4) during the 72 h of incubation was confirmed (Fig. 5A). Subsequently, the effect of cholesterol was tested. Since cholesterol is insoluble in aqueous solutions, albumin was used as a carrier. When fetal calf serum was replaced by pure albumin in the standard incubation medium, a decrease in α-tomatine in the cell-free medium was not observed during the incubation time. By contrast, >50% of α-tomatine was lost during the first 4 h of incubation when albumin containing the same concentration of cholesterol as the fetal calf serum (100 μM) was used. Therefore, interaction of α-tomatine with cholesterol appears to be the main reason for the decrease in α-tomatine in the medium during incubation, at least in the absence of cells.

To support this hypothesis, we also carried out tests to exclude the possible metabolism of α-tomatine caused by MCF-7 cells or enzymes present in the fetal calf serum added to an incubation medium. The incubation of 3 μM of α-tomatine in the standard incubation medium with and without MCF-7 cells led to a 60–70% loss of α-tomatine during the first 24 h. A comparable decrease in α-tomatine was observed when inactivated serum was added to the medium. Thus, the decrease is not due to biotransformation by plasma enzymes.

To screen the potential biotransformation by MCF-7 cells, a standard incubation medium with native fetal calf serum was used. Based on the α-tomatine structure, we anticipated that its biotransformation can proceed by deglycosidation or by oxidation (hydroxylation or N-oxidation) (24) of the aglycone. As these deglycosidated derivatives, with the exception of tomatidine, are not available on the market, acid hydrolysis of α-tomatine was carried out in order to get a reaction mixture containing compounds derived from α-tomatine by consecutive loss of sugar units. Hydrolysis products found in the reaction mixture are presented in Fig. 5B. Representative chromatograms for each compound were extracted from full scan data (Fig. 5B) and the retention times and masses (m/z) are presented in Table I.

Subsequently, samples of the incubation media and MCF-7 cell pellets after 4, 24, 48 and 72 h of incubation with 3 μM of α-tomatine were obtained and targeted metabolic screening for deglycosidated metabolites was carried out. Representative chromatograms (Fig. 5C) for metabolites possibly arising from deglycosidation were extracted from mass spectrometric data and compared with chromatograms of derivatives obtained by hydrolysis. Target metabolites were not found either in the incubation media or in the cell pellet. With this experiment, we proved that during incubation with an MCF-7 cell line, α-tomatine is not subjected to deglycosidation by biotransformation or by chemical decomposition.

Samples were also tested for the presence of oxidative biotransformation products (hydroxylation, N-oxidation) of α-tomatine and tomatidine. Since chemical standards of these metabolites are not available and chemical synthesis is problematic, the mass spectrometric data obtained was only inspected for masses of suspected mono-hydroxy (Δ m/z 16), di-hydroxy (Δ m/z 32) or N-oxide derivatives (Δ m/z 16). None of these suspected metabolites were found in the samples.

## Discussion

The mechanism of the cytotoxic action of α-tomatine has been studied intensively; nevertheless, the existing results differ significantly. In mouse EL4 lymphoma cells, α-tomatine at the concentration of 8 μM induces apoptosis with changes in the cell cycle distribution of these cells (53.8% sub-G1 peak) (19). In human prostatic adenocarcinoma PC-3 cells, a marked increase of apoptotic cells in sub-G1 phase (95%) was observed after treatment with 2 μM of α-tomatine for 24 h (18). The induction of apoptosis is closely related to the activation of cysteine proteases called caspases (25). In PC-3 cells, the activation of caspase-3, -8 and -9 in 2 μM of α-tomatine-treated cells was demonstrated (18). Contrary to these results, the induction of programmed cell death with no changes in caspase-3, -6, -7, -8 and -9 activity and with no changes in cell cycle distribution was demonstrated in α-tomatine-treated human leukemic cells HL60 and K562 (14) as well as MOLT-4 (20). Similarly, we demonstrated that caspase-8 and -9 are not activated. Our results indicate no changes in cell cycle distribution, no apoptotic sub-G1 peak in MCF-7 cells and no morphological signs of apoptosis after treatment with α-tomatine. α-Tomatine at the concentrations of 1–9 μM after 4 h induced neither SSBs nor DSBs detectable by comet assay in MCF-7 cells. Moreover, the activation of Chk2 related to DNA damage (26) was not detected. The tumor-suppressor phosphoprotein p53 plays a significant role in preventing inappropriate cell proliferation and maintaining genome integrity in response to genotoxic stress, resulting in cell-cycle arrest, DNA repair, apoptosis or senescence (27). Our experimental data show no changes in the level of p53 and p53 phosphorylated on serine 15 in MCF-7 cells after 4 and 24 h of α-tomatine treatment (1–6 μM). The levels of the cyclin-dependent kinase inhibitor p21^WAF1/CIP1^ also remained unchanged. Apoptosis is a process highly dependent on the content of intracellular ATP; there is an evident physiological difference in cells undergoing apoptosis or necrosis (28). We demonstrated a loss of ATP in α-tomatine-treated cells (6 and 9 μM) after 72 h, which supports the results of this study discussed above. A possible explanation of the cytotoxic mechanism could be the fact that cholesterol in biological membranes serves as a target for α-tomatine (see below).

A number of studies have indicated that α-tomatine inhibits the growth of various human cancer cell lines at low μM concentrations within 1–48 h of treatment (14–18,20,21). Our results showed a cytotoxic effect of α-tomatine at slightly higher concentrations than in the previous studies. We demonstrated that 6 and 9 μM of α-tomatine caused an inhibition of proliferation and a decrease in viability after 24 h in MCF-7 cells. However, after 48 and 72 h of α-tomatine treatment, a recovery of these cells occurred. Our study also revealed a transient increase and activation of check-point kinase 1 (Chk1) 4 h after α-tomatine application, which corresponds with the inhibition of proliferation. These changes in the levels of proteins disappeared after 24 h of incubation - an effect that corresponds with cell recovery. Chk1 is essential for cell viability and proliferation and for maintaining DNA integrity. Although it is active even in unperturbed cell cycles, it is further activated in response to DNA damage or stalled replication fork (26,29,30).

We investigated why the cells recovered after 24 h of incubation. In the course of that, we observed a decrease in the levels of α-tomatine solved in the media throughout the incubation. As this phenomenon occurred only when cholesterol was present in the media, the formation of an insoluble α-tomatine cholesterol complex (2) should be the main reason. It was reported (31,32) that α-tomatine is, in contrast to the related saponins, not interface seeking. The accumulation of α-tomatine at the water-air interface should therefore not be the cause of its potential disappearing from the solution.

Regarding biotransformation, fungal pathogens of tomato produce tomatinase enzymes which hydrolyze α-tomatine by removing the complete sugar moiety (β-lycotetraose) or partial sugars (9,24,33). Only aglycone (tomatidine) released is susceptible to hydroxylation by cytochrome P-450. Since the removal of even a single monosaccharide from the sugar moiety reduces the membranolytic activity of α-tomatine substantially (34), deglycosylation serves as a resistance mechanism of fungal pathogens. Our findings of no putative metabolites of α-tomatine, neither in the medium nor in cell pellets, are consistent with the current knowledge which suggests that mammalian cells do not metabolize α-tomatine.

Cholesterol in the membranes of MCF-7 cells probably served as a target for α-tomatine as well. The release of the cytosolic enzyme LDH which occurred especially at concentrations of α-tomatine 6 and 9 μM, is a marker of membrane integrity loss typical for early necrosis and only the late stage of apoptosis (35). The absence of any characteristic features of apoptosis, the morphological signs as well as the ATP depletion observed, rather indicate necrotic cell death. Although the rupture of membranes occurs in necrosis mediated by different mechanisms (36), the binding of α-tomatine to membrane cholesterol and the consequent membrane disruption may have had a triggering effect in the present study (37,38). Membrane disintegration by several other agents was shown to induce necrosis (35,39). Steroidal glycoalkaloids form complexes with free 3β-hydroxy sterols, such as cholesterol, present in the outer leaflet of the plasma and outer mitochondrial membranes of animal cells. A network of these complexes undergoes a lateral aggregation through sugar-sugar interactions between the sugar moieties, causing a rearrangement and subsequent loss of the barrier function. α-Tomatine has the strongest membrane-disruptive effect of all *Solanum* glycoalkaloids studied thus far (34,38).

In conclusion, our results show that a single application of α-tomatine has an antiproliferative effect on cancer cells within 24 h of incubation, but then the cells recover. Since α-tomatine is not biotransformed in the MCF-7 cell line via deglycosidation or oxidation during 72 h but the concentration of α-tomatine significantly decreases in solutions and media containing cholesterol, this effect is most probably related to the ability of α-tomatine to bind with cholesterol present in the culture medium. Our study is the first to describe this phenomenon, which should be taken into consideration when interpreting results from an *in vitro* assay with α-tomatine. α-Tomatine does not induce DNA damage or the activation of caspases; it does not change the levels of proteins p53 and p21 in the MCF-7 cell line but rather causes a decrease in the cellular ATP. These facts, along with the morphological changes observed, suggest that the decrease in viability of MCF-7 cells by α-tomatine is not due to apoptosis induction. A probable mechanism involved in the cytotoxicity is the membrane-disruptive effect due to its binding with membrane cholesterol.

## Figures and Tables

**Table I:** Retention times and m/z of pseudomolecular ions [M+H]^+^ of α-tomatine hydrolysis products.

Compound	t_r_ (min)	m/z
Tomatine	8.91	1034.5
GLC-GLC-GAL-TOD	8.76	902.5
XYL-GLC-GAL-TOD	9.52	872.5
GLC-GAL-TOD	9.47	740.5
GAL-TOD	8.91	578.5
Tomatidine (TOD)	17.62	416.4
